# Learning Spatial Aversion Is Sensory-Specific in the Hematophagous Insect *Rhodnius prolixus*

**DOI:** 10.3389/fpsyg.2018.00989

**Published:** 2018-07-09

**Authors:** Sebastian Minoli, Agustina Cano, Gina Pontes, Amorina Magallanes, Nahuel Roldán, Romina B. Barrozo

**Affiliations:** ^1^Laboratorio de Fisiología de Insectos, Instituto de Biodiversidad y Biología Experimental y Aplicada-CONICET, Buenos Aires, Argentina; ^2^Departamento de Biodiversidad y Biología Experimental-FCEN, Universidad de Buenos Aires, Buenos Aires, Argentina

**Keywords:** learning, sensory modalities, insects, triatomines, operant, aversive

## Abstract

Even though innate behaviors are essential for assuring quick responses to expected stimuli, experience-dependent behavioral plasticity confers an advantage when unexpected conditions arise. As being rigidly responsive to too many stimuli can be biologically expensive, adapting preferences to time-dependent relevant environmental conditions provide a cheaper and wider behavioral reactivity. According to their specific life habits, animals prioritize different sensory modalities to maximize environment exploitation. Besides, when mediating learning processes, the salience of a stimulus usually plays a relevant role in determining the intensity of an association. Then, sensory prioritization might reflect an heterogeneity in the cognitive abilities of an individual. Here, we analyze in the kissing bug *Rhodnius prolixus* if stimuli from different sensory modalities generate different cognitive capacities under an operant aversive paradigm. In a 2-choice walking arena, by registering the spatial distribution of insects over an experimental arena, we evaluated firstly the innate responses of bugs confronted to mechanical (rough substrate), visual (green light), thermal (32°C heated plate), hygric (humidified substrate), gustatory (sodium chloride), and olfactory (isobutyric acid) stimuli. In further experimental series bugs were submitted to an aversive operant conditioning by pairing each stimulus with a negative reinforcement. Subsequent tests allowed us to analyze if the innate behaviors were modulated by such previous aversive experience. In our experimental setup mechanical and visual stimuli were neutral, the thermal cue was attractive, and the hygric, gustatory and olfactory ones were innately aversive. After the aversive conditioning, responses to the mechanical, the visual, the hygric and the gustatory stimuli were modulated while responses to the thermal and the olfactory stimuli remained rigid. We present evidences that the spatial learning capacities of *R. prolixus* are dependent on the sensory modality of the conditioned stimulus, regardless their innate valence (i.e., neutral, attractive, or aversive). These differences might be given by the biological relevance of the stimuli and/or by evolutionary aspects of the life traits of this hematophagous insect.

## Introduction

As it happens in most animals, insects’ sensory systems can detect a wide range of stimuli but respond only to a few of them, usually the most relevant ones. This process of filtering irrelevant information is essential for any living being, which would otherwise be engaged in a continuous outcome of triggered behaviors belonging to different contexts. Moreover, according to their specific life habits, animals can prioritize the use of different sensory modalities to maximize the exploitation of available resources from the environment. For example, to find a food source some animals use mainly the visual system, while others make use mainly of their chemical senses (i.e., olfactory or gustatory). As a result, stimuli from different modality can be more or less significant for an individual. Usually these differences are reflected in the complexity of particular sensory structures of each species, which sometimes present remarkable specializations of associated sensory organs.

Besides, the set of stimuli to which an organism responds can change along its lifetime, and thus the same individual can stop responding to some and start responding to originally neutral stimuli. This behavioral plasticity can be induced by several factors, such as the nutritional and reproductive status, time of the day, previous experiences, among other. In anyway, regardless its physiological origin, behavioral plasticity allows animals to maximize the efficiency of exploitation of unstable and/or unpredicted environments by allowing animals to modulate their responses according to immediate needs.

In particular, experience dependent plasticity allows animals to finely tune innate responses and even to respond to stimuli that being originally neutral gain certain relevancy after a reinforced experience. It is not surprising then that learning capacity has been revealed in almost all studied animals. In this sense, adapting preferences to time-dependent relevant environmental conditions provide a wider and cheaper behavioral reactivity. Learning involves a complex series of processes that promote reversible modifications in particular behaviors which can be highly adaptive, generating a memory of that event. Two main types of learning have been well described so far: non-associative and associative. The first one is generated after the repetition of a unique type of stimulus that, without any reinforcement increases (sensitization) or decreases (habituation) the intensity and/or frequency of the subsequent response of the individual to the same stimulus (Kandel, 1991; Rakitin et al., 1991; Menzel, 1999). The second is the process by which an association between two stimuli or a behavior and a stimulus is formed, if properly reinforced (Bitterman et al., 1983; Menzel and Muller, 1996). Two main forms of associative learning have been described in animals. In Pavlov’s classical conditioning (Pavlov, 1929) a previously neutral stimulus is repeatedly presented together with a reflex-eliciting stimuli followed by a reinforcement, until eventually the neutral stimulus will elicit a response on its own. In Skinner’s operant conditioning (Skinner, 1937) a certain behavior is followed by a reinforcement, resulting in an altered probability that the behavior will happen again. Associative forms of learning allow individuals to anticipate events by recognizing marks previously related to them. In this work we designed and applied an associative operant aversive conditioning.

Learning abilities can largely differ across species, individuals and even throughout lifespan and can be modulated by several features of the training procedures (Menzel et al., 2001; Deisig et al., 2007; Giurfa and Sandoz, 2012; Giurfa, 2015). Among them, the salience of the conditioned stimulus has a relevant role in determining the intensity of an association (Menzel and Muller, 1996). As a generality, salient stimuli are more prone to generate a conditioned response than those that do not differ much from the environmental basal sensory information. Thus, given that animals prioritize different sensory modalities according to their habits (e.g., diurnal animals usually make use of visual cues while nocturnal ones do not), the cognitive abilities of an individual might be reflected in these differences. We analyze in this work if stimuli from different sensory modality can generate different cognitive performances in kissing bugs.

*Rhodnius prolixus* (Heteroptera: Reduviidae: Triatominae) is an hematophagous insect, vector of the Chagas disease in Latin America. Up to date, there are no vaccines that can prevent the transmission of the *Trypanosoma cruzi*, parasite responsible for this illness in humans. This fact intensifies the relevance of studying this vector of a human disease, as adding knowledge about its physiology, behavior and/or ecology permits to increase the general knowledge about this species and at the same time can help in improving the efficiency of field control strategies. In fact, since Wigglesworth and Gillett’s (1934) pioneer works this blood-sucking bug has classically been an experimental model in the study of physiology of behavior in insects. However, it was only few years ago that their learning capacities have captured the attention of researchers. Vinauger et al. (2011a,b) applied a classical conditioning approach and succeeded in training *R. prolixus* to associate lactic acid (a neutral odor) with food (i.e., positive reinforcement) or with a mechanical perturbation (i.e., negative reinforcement). They found that in further tests, *R. prolixus* walked toward or against the lactic acid, respectively. Moreover, even if *R. prolixus* did not present a preference in a walking olfactometer when odors from a live rat and quail were presented simultaneously at opposite sides, an aversive conditioning generated an aversion to one or the other host according to the training procedure (Vinauger et al., 2012). In addition, kissing bugs extend their proboscis (PER, for proboscis extension response) when they perceive a warm object at the correct temperature and distance. Taking advantage of this unconditioned response, Vinauger et al. (2013) demonstrated that the PER of *R. prolixus* can be modulated by non-associative and associative learning forms. In a completely different context, Minoli et al. (2013) showed that the innate escape response of kissing bugs to the alarm pheromone can be widely modulated by associative and non-associative conditioning protocols. Moreover, it was reported for the same species that a brief pre-exposure to bitter compounds prevents insects from feeding on an appetitive solution (Pontes et al., 2014). Later, triatomines’ cognitive abilities showed to follow a circadian rhythm (Vinauger and Lazzari, 2015). These authors describe that bugs perform well during the night, but not during the day. Studying the repellent effect of new non-toxic molecules for *R. prolixus*, Asparch et al. (2016) found that bugs are innately repelled by different bitter molecules, and that this repellence can be modulated by associative and non-associative forms of learning. Indeed, after an aversive operant conditioning, bugs’ behavior changed from avoidance to indifference or even to preference, according with the protocol applied (Asparch et al., 2016). In another work, Mengoni et al. (2017) studied the experience-dependent plasticity of the innate attractive response of kissing bugs to feces. These authors describe that after pre-exposing bugs to feces for 24 h, insects were no longer attracted to feces. Finally, by pairing the presence of feces with an aversive mechanical disturbance, nymphs switched from attraction to avoidance of feces.

In this work we addressed the question whether stimuli from different sensory modality can generate different learning performances under an operant aversive protocol. We firstly studied the innate responses of *R. prolixus* to mechanical, visual, thermal, hygric, gustatory, and olfactory stimuli. Then we analyzed if such responses can be modulated by an operant aversive conditioning. Stimuli could be innately neutral, attractive or aversive and change their perceptual value after training. We discuss possible roles of the modality and/or type of stimulus in the efficiency of the learning process and its relation with the biological relevance of the stimulus.

## Materials and Methods

### Insects

*R. prolixus* was reared in an insectary at 28 ± 1°C, 40 ± 10% relative humidity and an L:D 12:12 h inverted photoperiod cycle. Each week, newly emerged fifth instar nymphs were collected from the rearing chamber and maintained unfed for 7–15 days prior to their use in experiments. This is a moderate starving status since once fed, these hematophagous insects can resist up to 60 days without feeding again. Insects were used only once and then discarded. A total of 540 insects were used along this work. All experiments were carried out in functional darkness during the first 6 h of their scotophase (i.e., 0–6 h after lights were turned-off) as to match the maximal activity period described for triatomines (Lazzari, 1992) and at the same time to exclude external visual cues. The temperature of the experimental room was set to 25 ± 1°C before the beginning of each assay and the relative humidity ranged between 30 and 60%.

In order to minimize potential effects of inbreeding, our insectary is frequently provided with new insects by the Servicio Nacional de Chagas (Santa María de Punilla, Córdoba, Argentina). All animals were handled according to the biosafety rules of the Hygiene and Safety Service of the Universidad de Buenos Aires.

### Two-Choice Walking Arena

**FIGURE 1:**
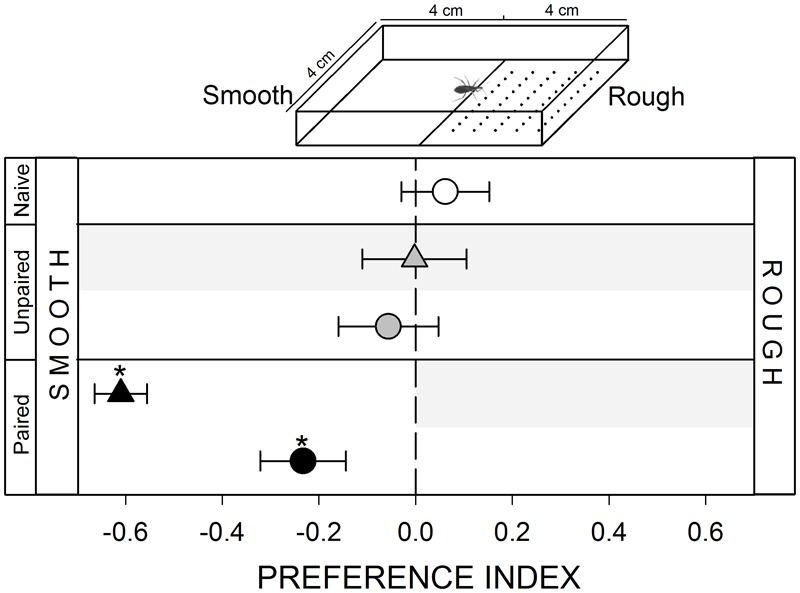
Responses of *Rhodnius prolixus* to a mechanical stimulus. No innate response was registered when insects were confronted to a smooth/rough substrate choice (white circle, *p* > 0.05). Unpaired yoke controls presented the same pattern: i.e., no preference (gray triangle and circle, *p* > 0.05 in both cases). During training, insects avoided the punished zone: i.e., the rough side (black triangle, *p* < 0.05). During test the smooth side was still preferred (black circle, *p* < 0.05) evincing an experience-dependent behavioral plasticity. Each point represents the mean (±SE) spatial preference of 30 insects individually released in a 2-choice rectangular walking arena. Asterisks denote statistical differences between the PI and the value 0 (*p* < 0.05) evinced by One sample *T*-test. Gray shadows show the zone in which punishment was delivered during unpaired and paired trainings.

**FIGURE 2:**
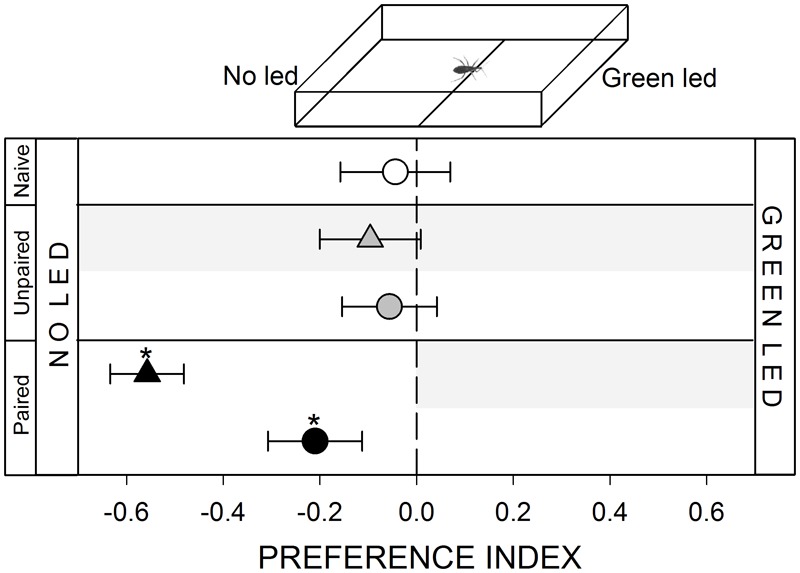
Responses of *R. prolixus* to a visual stimulus. No innate response was registered when insects were confronted to a “no led/green led” choice (white circle, *p* > 0.05). Unpaired yoke controls presented the same pattern: i.e., no preference (gray triangle and circle, *p* > 0.05 in both cases). During training, insects avoided the punished zone: i.e., the “green led” side (black triangle, *p* < 0.05). During test the no-led side was still preferred (black circle, *p* < 0.05) evincing an experience-dependent behavioral plasticity. Each point represents the mean (±SE) spatial preference of 30 insects individually released in a 2-choice rectangular walking arena. Asterisks denote statistical differences between the Preference Index (PI) and the value 0 (*p* < 0.05) evinced by One sample *T*-test. Gray shadows show the zone in which punishment was delivered during unpaired and paired trainings.

**FIGURE 3:**
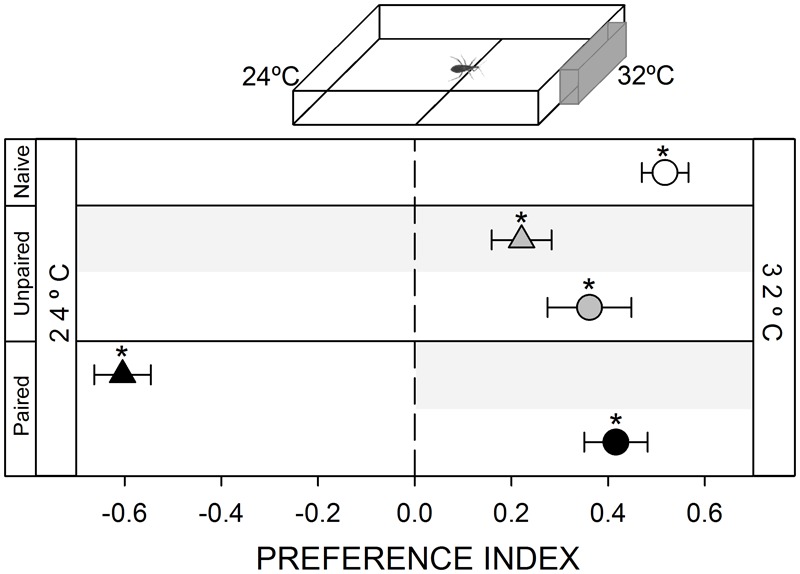
Responses of *R. prolixus* to a thermal stimulus. A 32°C heated plate was attractive for naïve insects (white circle, *p* < 0.05). Unpaired yoke controls presented the same pattern: i.e., attraction to heat (gray triangle and circle, *p* < 0.05 in both cases). During training, insects avoided the punished zone: i.e., the hot side (black triangle, *p* < 0.05). During test insects continued to prefer the hot side (black circle, *p* < 0.05), evincing that this attraction was not modulated by the aversive conditioning. Each point represents the mean (±SE) spatial preference of 30 insects individually released in a 2-choice rectangular walking arena. Asterisks denote statistical differences between the PI and the value 0 (*p* < 0.05) evinced by One sample *T*-test. Gray shadows show the zone in which punishment was delivered during unpaired and paired trainings.

**FIGURE 4:**
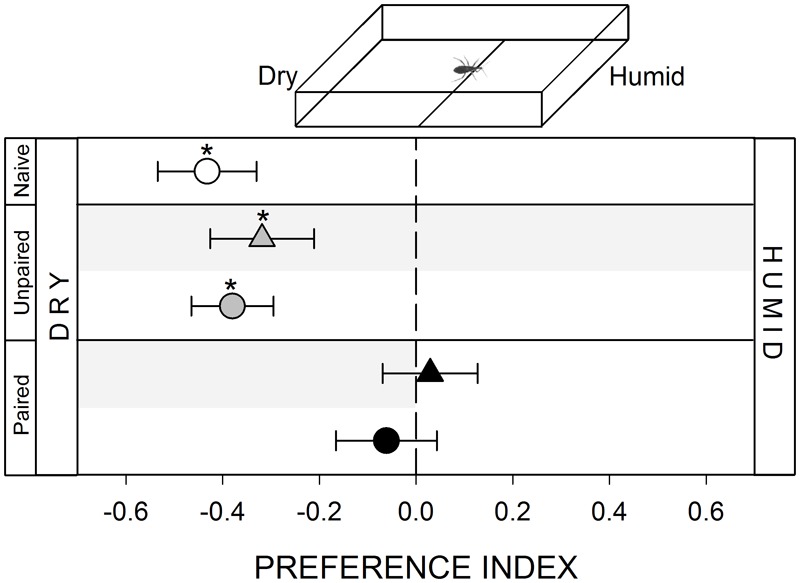
Responses of *R. prolixus* to a hygric stimulus. Insects innately avoided the humid zone of the arena (white circle, *p* < 0.05). Unpaired yoke controls presented the same pattern: i.e., hygric avoidance (gray triangle and circle, *p* < 0.05 in both cases). However, this avoidance disappeared during (black triangle, *p* < 0.05) and after (black circle, *p* > 0.05) training, evincing a partial modulation of this avoidance. Each point represents the mean (±SE) spatial preference of 30 insects individually released in a 2-choice rectangular walking arena. Asterisks denote statistical differences between the PI and the value 0 (*p* < 0.05) evinced by One sample *T*-test. Gray shadows show the zone in which punishment was delivered during unpaired and paired trainings.

**FIGURE 5:**
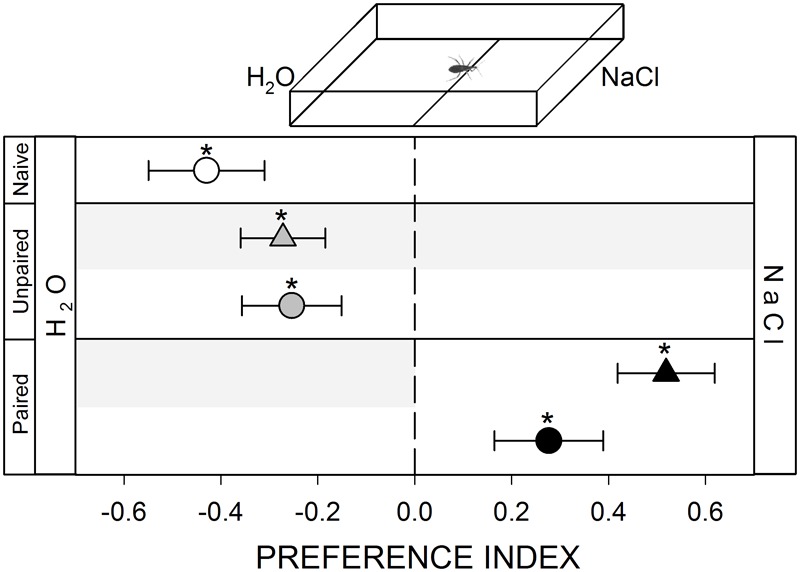
Responses of *R. prolixus* to a gustatory stimulus. Insects were innately repelled by NaCl (white circle, *p* < 0.05). Yoke controls presented the same pattern (gray triangle and circle, *p* < 0.05 in both cases). During training, insects avoided the punished zone: i.e., the H_2_O side (black triangle, *p* < 0.05). During test NaCl side was still preferred (black circle, *p* < 0.05) evincing an experience-dependent behavioral plasticity. Each point represents the mean (±SE) spatial preference of 30 insects individually released in a 2-choice rectangular walking arena. Asterisks denote statistical differences between the PI and the value 0 (*p* < 0.05) evinced by One sample *T*-test. Gray shadows show the zone in which punishment was delivered during unpaired and paired trainings.

**FIGURE 6:**
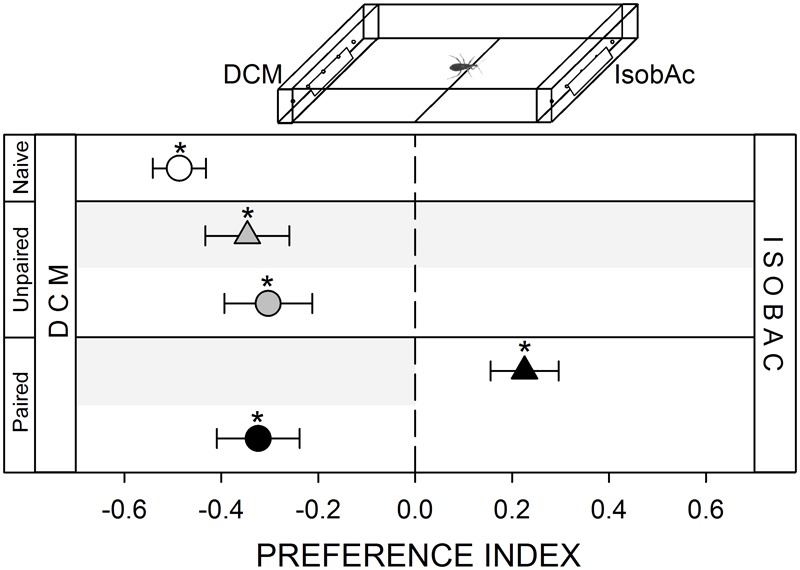
Responses of *R. prolixus* to an olfactory stimulus. IsobAc was repellent for these insects (white circle, *p* < 0.05). Yoke controls presented the same pattern (gray triangle and circle, *p* < 0.05 in both cases). During training, insects avoided the punished zone: i.e., the DCM side (black triangle, *p* < 0.05). During test insects continued to avoid the IsobAc side (black circle, *p* < 0.05), evincing that the repellence could not be modulated by the aversive conditioning. Each point represents the mean (±SE) spatial preference of 30 insects individually released in a 2-choice rectangular walking arena. Asterisks denote statistical differences between the PI and the value 0 (*p* < 0.05) evinced by One sample *T*-test. Gray shadows show the zone in which punishment was delivered during unpaired and paired trainings.

To study the responses of *R. prolixus* to stimuli of different modality, insects were individually released at the center of a walking rectangular acrylic arena of 8 cm × 4 cm, virtually divided by a line in two equal zones of 4 cm × 4 cm (see inset of **Figures 1–6**). According to the experimental series, a particular stimulus was added at one zone of the arena while the opposite zone was maintained as the corresponding control zone. To facilitate the walking behavior of bugs, the floor of the arena was covered with filter paper, which was replaced between replicates to avoid chemical contamination among assays. To avoid spatial heterogeneities other than those intentionally added, the position of the stimuli was switched between left and right side in a pseudorandom manner (i.e., 15 times at each side).

Stimuli from different modalities were tested, i.e., mechanical, visual, thermal, hygric, gustatory, and olfactory. In all cases, once the stimuli were settled and stabilized over the arena, one insect was gently released at its center and left to freely walk during 4 min. During this experimental time, its spatial distribution in relation to the position of the stimulus (e.g., attraction, repellence or indifference) was recorded in video using an infrared-sensitive video-camera connected to a digital recorder. The time spent at each zone of the arena was then obtained from the video films and a preference index (PI) was calculated for each individual as the difference between the time spent at the stimulus zone (*Ts*) minus the time spent at the control zone (*Tc*) divided by the total experimental time:

$$PI=\frac{TS-TC}{TS+TC}$$

PIs near 0 indicate lack of preference (neutral stimulus); PIs close to -1 show preference for the control zone (repellent stimulus); PIs close to 1 show preference for the stimulus zone (attractive stimulus).

### Stimuli and Modalities

Responses of *R. prolixus* to six stimuli of different modality were analyzed in the two-choice walking arena. Although in each case the addition of the stimulus and its corresponding control was accomplished differently, the goal was always the same: generate a spatial heterogeneity in the arena for *R. prolixus*. We then analyzed if such spatial heterogeneity evoked an innate response in insects and if such responses could be modulated by a previous experience.

#### Mechanical Stimulus

A mechanical stimulus was added in the arena by making multiple holes (≅ 1 mm diameter, ≅ 1 mm distance between holes) with an awl to the filter paper covering the floor of the stimulus zone (inset **Figure 1**). Previous experiments performed in our laboratory show that *R. prolixus* can detect this roughness in the substrate during walking (unpublished data). The control zone was maintained intact generating a “smooth/rough” spatial heterogeneity.

#### Visual Stimulus

A green led (5 mm diameter, 2.4 V, 520–550 nm) controlled with a dimmer was added outside the arena, 2 cm away from the distal wall of the stimulus zone (inset **Figure 2**). Light could pass through the transparent acrylic wall of the arena and reach the position of insects. Previous works show that *R. prolixus* can perceive green light (Reisenman et al., 2000). The low intensity chosen (1 ± 0.2 lux) allowed us to offer a punctual visual cue that barely illuminated the arena. No light was added at control zone.

#### Thermal Stimulus

A thermal heterogeneity was generated in the arena by heating the wall at the end of the stimulus zone by contacting it externally with a thermostatized heated plate (inset **Figure 3**). A layer of thermal grease was added between both surfaces to improve thermal conduction. In this way, temperature in the inner side of the acrylic wall of the stimulus zone was stabilized at 32 ± 0.5°C, while the inner wall of the control zone was maintained at ambient temperature, i.e., 24 ± 0.5°C. Temperature was chosen as to match skin temperature of triatomines natural hosts.

#### Hygric Stimulus

To generate an hygric heterogeneity in the arena we added 100 μl of distilled water on the filter paper covering the stimulus zone. A micropipette allowed us to distribute the water homogeneously (inset **Figure 4**). Volume added was chosen as to make sure filter paper was wet but did not present puddles. In this way, this zone of the arena was humid, while the control zone was maintained dry. The experiment started immediately after loading the water (i.e., 1 min approximately) in order to minimize water evaporation.

#### Gustatory Stimulus

To generate a gustatory heterogeneity in the arena we added (homogeneously with a micropipette) 100 μl of 1 M NaCl over the filter paper covering the floor of the stimulus zone and 100 μl of distilled water on the control zone (inset **Figure 5**). The NaCl (purchased in Biopack, Argentina) solution was prepared in distilled water. This concentration was chosen as in previous works it was efficient in deterring feeding in the same species (unpublished data). The experiment started immediately after loading the water and the NaCl solution (i.e., 1 min approximately) in order to minimize water evaporation.

#### Olfactory Stimulus

An olfactory gradient was generated over the arena by adding IsobAc at the stimulus zone and DCM, (solvent used to dilute IsobAc) at the control zone. Previous works show that this odorant generates an escape response in this species (Cruz-López et al., 2001; Minoli et al., 2013). The experimental arena was slightly adapted for this series by performing five holes (1 mm diameter) at the bottom of the distal walls of each zone (inset **Figure 6**). Outside these walls, a chamber containing IsobAc communicated with the interior of the arena via the holes. The addition of the odorant was achieved by placing a piece of filter paper (2 × 1 cm) loaded with 1000 μg of IsobAc dissolved in 20 μl of DCM in one chamber and another paper loaded with 20 μl of DCM in the opposite chamber. In this way, vapors released by the papers entered the arena through the holes and generated a chemical gradient. IsobAc and DCM were purchased from Sigma-Aldrich (St. Louis, MO, United States).

### Operant Aversive Conditioning

To analyze if the responses of *R. prolixus* to different stimuli are differentially modulated by a previous experience, we applied an operant aversive conditioning and analyze if the insects’ innate preferences were modulated or not. For this purpose we used the same experimental arena described above but with the addition of a vortex mixer (40 Hz) that, being in contact with the base of the arena, allowed us to generate a vibration that reached the insects via the substrate and was applied as negative reinforcement. This vibration was shown to be innately aversive for *R. prolixus* (Minoli et al., 2013) and could be voluntarily controlled by the manipulator via a manual switch. Before experiments and for each experimental series we predefined if the stimulus zone or the control zone were associated with the negative reinforcement according to the innate responses of the insects (see **Table 1**). Innately attractive stimuli were positioned at the punishment side and aversive ones at the safe side. This decision was assumed as to intend to turn over the innate valence of the stimulus with the aversive conditioning. Neutral stimuli were arbitrarily placed at the punishment side.

**Table 1:** Stimuli associated with the safe and the punishment side of the arena in each experimental series.

Series	Stimulus at the safe zone	Stimulus at the punishment zone
Mechanical	Smooth	Rough
Visual	No led	Green led
Thermal	24°C	32°C
Hygric	Humid	Dry
Gustatory	NaCl	Solvent
Olfactory	IsobAc	Solvent

In this way, for each sensory modality, a 4 min training period was applied in which the negative reinforcement was applied to the insects whenever they entered the predefined punishment zone. The vibration ended as soon as the insect entered the safe zone of the arena. Yoke control series were run in parallel in which each individual received the negative reinforcement independently from its position in the experimental arena. The timing, frequency and duration of the vibration were copied from the previously conditioned insect.

The behavior of each individual during training time was registered in video and the individual PIs were computed. Once training ended, the bug was removed from the arena and released in an individual flask for 1 min. Following this time, it was transferred to the two-choice arena where its preference was tested as explain in Section “Two-Choice Walking Arena.” Note that separated PIs were registered for training (triangles in the figures) and test (circles).

### Data Analysis and Statistics

The PI of each individual was computed. For each experimental series (i.e., mechanical, visual, thermal, hygric, gustatory, and olfactory series), thirty individuals were tested in each group (i.e., 30 naïve, 30 yoke control and 30 paired), totalizing 540 insects. Insects were used only once and then discarded. The mean PI of each series was compared against the expected value if there were no preferences, i.e., “0.” One-sample *T*-tests were applied to statistically assess this difference (Sokal and Rohlf, 1995). Normality and homoscedasticity of data were checked. All figures represent the mean PIs (*x*-axis) and standard errors, and the stimuli presented at each zone of the arena (*y*-axis).

## Results

Innate responses of *R. prolixus* in the 2-choice walking arena varied among stimuli (see **Figures 1–6**, white circles). The rough substrate (mechanical stimulus) and the green led (visual stimulus) were neutral, i.e., they did not modify bugs’ distribution over the arena. As expected, the heated plate (thermal stimulus) generated an innate attraction. Conversely, the distilled water (hygric stimulus), the NaCl (gustatory stimulus) and the IsobAc (olfactory stimulus) were innately repellent. The experience-dependent plasticity of the responses of *R. prolixus* varied according to the stimulus and is dissected in the next section.

### Innate Responses and Experience-Dependent Modulation

#### Lack of Response to a Textured Substrate

Kissing bugs are thigmotactic animals, i.e., they try to maintain physical contact with objects that provide a mechanical stimulus. In their natural environments they remain a great part of the day in contact with different materials from their shelters and with conspecifics. In our experiments, the addition of a rough substrate in the experimental arena did not generate a preference in bugs (**Figure 1**, white circle, *p* > 0.05). However, the vibration caused by the vortex mixer was clearly perceived as a negative stimulus for bugs, as during training they avoided the punishment zone (**Figure 1**, black triangle, *p* < 0.05), i.e., the rough substrate. During the test, in which negative reinforcements were no longer delivered, this avoidance for the zone containing the rough substrate was still expressed (**Figure 1**, black circle, *p* < 0.05), demonstrating that bugs established an association between the physical properties of the substrate and the occurrence of a punishment.

#### Lack of Response to a Punctual Green Light

Previous works show that kissing bugs avoid ambient light (Reisenman and Lazzari, 2006) but can be attracted to low intensity punctual light sources (Minoli and Lazzari, 2006). In the present work, a punctual green light source at one side of the arena was neither attractive nor repulsive for *R. prolixus* (**Figure 2**, white circle, *p* > 0.05). Like in the previous series, the negative reinforcement applied at the green led zone caused a spatial preference for the opposite side of the arena (**Figure 2**, black triangle, *p* < 0.05). During the posterior test, insects continued to avoid the green led zone, demonstrating that insects could associate the visual stimulus with the punishment (**Figure 2**, black circle, *p* < 0.05).

#### Attraction to Heat

Thermal stimulation is among the most informative cues used by hematophagous insects to find a host (Lazzari and Nuñez, 1989; Lazzari, 2009). In our experimental setup, the addition of a hot plate at one side of the arena produced the highest attraction response registered in this work (**Figure 3**, white circle, *p* < 0.05). However, when the vibration was applied at the hot zone, bugs avoided it, evincing that the negative value of the vibration is somehow more intense than the positive value of the heat *per se* (**Figure 3**, black triangle, *p* < 0.05). However, in this case, differently from previous series, during the posterior test bugs preferred to occupy the heated side just as naïve insects, indicating that the association between heat and the vibration could not be established or was not expressed (**Figure 3**, black circle, *p* < 0.05).

#### Avoidance of a Wet Substrate

The presence of distilled water over the walking substrate produced an avoidance behavior in bugs (**Figure 4**, white circle, *p* < 0.05). Previous studies have shown that kissing bugs present marked humidity preferences (Roca and Lazzari, 1994; Lorenzo and Lazzari, 1998; Guarneri et al., 2002). In this work we show for the first time the existence of an aversion for wet substrates in kissing bugs. Surprisingly, during the conditioning period in which the dry zone of the arena was defined as the punishment zone, insects spent half of the time at each zone (**Figure 4**, black triangle, *p* > 0.05). This was the only series along this work in which the punishment zone was not avoided during conditioning, suggesting that bugs perceived the wet substrate as negative as the negative reinforcement. However, during the posterior test bugs continued to exhibit a random distribution (**Figure 4**, black circle, *p* > 0.05), evincing at least a partial modulation of the innate behavior of avoiding wet substrates.

#### Salt Repellence

Once a kissing bug reaches the skin of a potential host, their gustatory sense starts to play a relevant role in its feeding decision. Previous works show that *R. prolixus* can identify aversive and/or appetitive molecules that will deter or induce the feeding process (Pontes et al., 2014, 2017). We show here that *R. prolixus* avoids walking in zones containing high concentrations of NaCl (**Figure 5**, white circle, *p* < 0.05). Just as in previous series (except in the hygric one), during training bugs avoided the punishment zone even if they had to remain in the aversive zone (**Figure 5**, black triangle, *p* < 0.05). In the posterior test, insects continued to avoid the punishment zone, even if vibrations were no longer delivered, preferring to stay at the NaCl zone (**Figure 5**, black circle, *p* < 0.05). This result shows an experience-dependent modulation of their gustatory preference.

#### Avoidance of the Alarm Pheromone

Adult kissing bugs release IsobAc as the main component of an alarm pheromone, and nymphs and adults are repelled by this signal (Manrique et al., 2006). In our setup, *R. prolixus* innately avoided the zone of the arena containing IsobAc (**Figure 6**, white circle, *p* < 0.05). During training, bugs avoided the punishment side, remaining mostly in the IsobAc zone (**Figure 6**, black triangle, *p* < 0.05). In the posterior test, bugs avoided the IsobAc (**Figure 6**, black circle, *p* < 0.05), evincing that they were either not able to generate an association between the olfactory stimulus and the occurrence of the punishment or that they couldn’t express it.

#### Yoke Control: Unpaired Delivery of the Negative Reinforcement

In all yoke series, the random delivery of vibration did not affect the expression of the innate behavior of insects. During both, trainings and test, yoke control insects behaved as naïve ones, i.e., a random behavior when confronted to mechanical (**Figure 1**, gray triangle and circle, *p* > 0.05 in both cases) and visual stimuli (**Figure 2**, gray triangle and circle, *p* > 0.05 in both cases), an attraction to the heated side of the arena (**Figure 3**, gray triangle and circle, *p* < 0.05 in both cases) and an aversion for hygric (**Figure 4**, gray triangle and circle, *p* < 0.05 in both cases), gustatory (**Figure 5**, gray triangle and circle, *p* < 0.05 in both cases) and olfactory stimuli (**Figure 6**, gray triangle and circle, *p* < 0.05 in both cases). These results confirm that in the conditioning series presented above, an associative learning was responsible for the modulation observed.

## Discussion

Learning is crucial to maximize the exploitation of resources in unpredictable environments. However, although it is expressed in most animals, it has been widely shown that small changes in the acquisition protocols can drastically modulate the efficiency of learning at different levels. In this work we studied how the sensory modality of the stimuli involved in the conditioning process can be a key factor for the correct acquisition of information from the environment. For this purpose, we maintained a unique operant aversive protocol, being the conditioned stimulus the only parameter that varied between experimental series. We then analyzed and compared if the responses to such stimuli were more or less prone to be modulated by such a previous experience.

Along our experiments, the mechanical vibration showed to be an efficient negative reinforcement for *R. prolixus*. During training of the two neutral series (i.e., mechanical and visual) insects avoided the punishment zone of the arena, showing that the vibration is indeed perceived by bugs as an aversive stimulus that generates a spatial avoidance (**Figures 1, 2**, black triangles). In the next four series (i.e., thermal, hygric, gustatory, and olfactory), in which an innate behavior was provoked by the conditioned stimuli, the punishment side was intentionally defined as to match the innately preferred zone of the arena. During the training of the thermal series, insects preferred the not-heated/safe zone of the arena rather than the heated/punishment zone (**Figure 3**, black triangles), evincing that the value of the negative reinforcement was higher than the positive attractive value of the heat. On the other hand, in training phases of innately repellent stimuli insects either lost their innate stimuli avoidance (i.e., hygric, **Figure 4**, black triangle), or they inverted it (i.e., gustatory and olfactory, **Figures 5, 6**, black triangles), evincing in this case that the negative value of the vibration was higher than that of the aversive stimuli. Being choice experiments, the expression of the spatial preference of the insects for one or the other side of the arena merely reflects a relative preference, and does not allow us to discern if it is the result of an attraction to the preferred side, a repellency for the avoided side, or if of both processes are acting together. Besides, it is worth noting that even if the vibration was clearly perceived as an aversive stimulus, the avoidance generated during training phases does not imply that the animals are able to modulate their behavior in an associative-dependent manner. For example, vibration was indubitably aversive for kissing bugs during the thermal series training, but during subsequent test they continued to be attracted to heat, suggesting that the association between heat/punishment was either not achieved or could not be expressed.

As expected, some of the stimuli triggered conspicuous innate responses in these insects. Heat, known to be among the most important cues in host finding for *R. prolixus* (Lazzari, 2009), was attractive for *R. prolixus*. Contrarily, the addition of IsobAc to the two-choice arena generated an innate repellence. IsobAc is the main component of the alarm pheromone of these insects and is a powerful activator and repellent (Cruz-López et al., 2001; Rojas et al., 2002; Manrique et al., 2006). It is not surprising then that these two intense responses belonging to two different but biologically relevant contexts (i.e., feeding and escaping from danger, respectively) were not modulated by the previous experience. Whereas behavioral plasticity might be a key process in fluctuant environments, innate and rigid responses are probably more adaptive if stable and honest stimuli are involved. In this sense, we can speculate about the possibility that responses to biologically relevant stimuli are less prone to be modulated by a previous experience. In this sense, learning to “not approach” a heat source and/or to stop avoiding the alarm pheromone might result in death by starvation or by being eaten by a potential predator.

Conversely, the innate avoidance of NaCl was effectively modulated by an aversive conditioning. In natural conditions, *R. prolixus* exerts a chemical scanning of the potential host skin using gustatory receptors present in their antennae. High levels of NaCl over the skin were shown to inhibit feeding of this species (Pontes et al., 2014). Accordingly, our results show that bugs prefer to avoid walking over substrates containing NaCl. However, following conditioning, bugs radically changed their behavior, even preferring to walk over the NaCl-loaded substrate rather than to do it in the control side. This is clear evidence that *R. prolixus* can learn from their previous experience in an aversive operant paradigm. Compared to the thermal and the olfactory cues discussed in the previous paragraph, learning to stop avoiding salty substrates might not have deleterious consequences.

Even if the visual spectrum and the negative phototaxia of kissing bugs have been quite well studied when ambient light is presented (reviewed in Barrozo et al., 2016), far less is known about the responses of these bugs to dimmed punctual light sources. Light traps have been reported to capture triatomines, but in low quantities (Vazquez-Prokopec et al., 2004; Carbajal de la Fuente et al., 2007). In an indoor flying cage, Minoli and Lazzari (2006) found that adult *R. prolixus* and *Triatoma infestans* initiate flight toward a white or an UV light source. Our results show that *R. prolixus* exhibits a random walking behavior in presence of the green led. However, this lack of response was modulated by the applied aversive conditioning, as insects learned to keep away from the green light to avoid punishment. Similarly, no behavioral preference was registered when different roughness in the substrate was offered to insects. Then, insects started to avoid the rough surface after the conditioning period. Differently from previous series, both the visual and the mechanical cues were neutral for bugs prior to the conditioning. However, animals learned to avoid the negative reinforcement by changing their spatial preference over the arena. Following the previous idea, those behaviors that do not seriously compromise the animal’s survival seem to be more easily modulated than those that might do it.

Several studies have shown that ambient humidity plays a relevant role in kissing bugs’ distribution (Roca and Lazzari, 1994; Lorenzo and Lazzari, 1999) and host finding (Barrozo et al., 2003). However, no previous data are available about the effect that a humid substrate might have on their walking behavior. We show here for the first time that *R. prolixus* avoids walking over a wet filter paper. Surprisingly, during conditioning, insects exhibited a random occupancy of each zone of the arena. This is the only series in which bugs did not avoid the punishment zone during training. This result could be attributable to a similarity in the perceived negative value of the vibration (i.e., the negative reinforcement) and the wet substrate. However, the effect of the conditioning became evident during the test, as the innate avoidance of the humid zone vanished, remaining bugs similar amount of time at each zone. This result shows that *R. prolixus* is able to modulate its hygric avoidance behavior after an aversive operant conditioning, although the intensity of the modulation seems to be low.

Different parameters of a learning protocol can modulate its efficiency (Menzel et al., 2001; Deisig et al., 2007; Giurfa and Sandoz, 2012; Giurfa, 2015). Among them, it has been shown that massed- and spaced-trials conditionings favor short and long term memories, respectively. As well, the intensity of the acquisition process can be modulated by the timing of the contingency between the conditioned stimulus (CS) and the unconditioned stimulus (US). The phase, duration and frequency of the paired presentation of the CS and the US play an important role in the acquisition efficiency. Besides, as a general rule, the higher the salience of a particular stimulus, the better the learning score (Menzel and Muller, 1996). However, these are just few of the relevant factors that can modulate the learning capacity of an individual. In our work, we describe how different stimuli can generate differences in the learning capacities of an animal. Maintaining the same operant protocol (i.e., same training time, same time between training and test, same negative reinforcement, same experimental device, etc) we show that the quality of the stimulus used as CS is a key factor for the efficiency of the learning process. However, being that our experiments were performed under an operant protocol design, the intensity of the negative reinforcement could only be determined by the behavior of each individual (remember that the occurrence of the vibration was determined by the position of each insect in the experimental arena). The number of vibrations (**Supplementary Figure S1A**) and the vibration time (**Supplementary Figure S1B**) varied across experimental series (One-way Anova, *p* < 0.05 in both cases, statistical differences after Tukey’s *post hoc* comparisons showed in letters in the figures). However, no statistical correlations between any of these two parameters of training and the learning performances were obtained (**Supplementary Figure S2**, *p* > 0.05 for all correlations, *R*^2^ showed in the figure). These results suggest that the sensory modality of the conditioned stimulus is the main parameter controlling the efficiency of our aversive conditioning paradigm.

As a general observation, our results allow us to speculate about the possibility that animals can modulate their innate responses more easily if the conditioned response is not directly or indirectly harmful for the individual. In this sense, the attraction toward a heat source is an evolutionary conserved behavior that resisted the conditioning designed and applied during this work. The thermal sense of these bugs is probably the most important input implicated in detecting a potential host (Lazzari and Nuñez, 1989; Ferreira et al., 2007). The inhibition of this behavior would then interfere directly in the feeding process, reason why the modulation of this behavior is probably blocked. In fact, thermal experiments carried out in this work were performed using intermediately starved animals (i.e., 7–15 days). Further experiments using recently fed bugs could help in confirming this hypothesis. Previous studies have shown that the proboscis extension response of *R. prolixus* in response to a heat source can indeed be negatively modulated by an aversive conditioning (Vinauger et al., 2013; Vinauger and Lazzari, 2015). However, although *a priori* our results and those presented by Vinauger and collaborators seem to be contradictory, they are instead complementary, as different moments of the feeding process are analyzed in each case. While we registered the approach behavior to the potential food source, Vinauger and collaborators studied the extension of the proboscis to start feeding. It seems then that different phases of the feeding behavior of *R. prolixus* can be differentially modulated by previous experience. Similarly, the conditioning protocol did not succeed in modulating the avoidance response of these bugs to IsobAc. Adults of this species release an alarm pheromone when a potential danger is near. If kissing bugs were to stop escaping from this cue, their lives would probably be endangered. It is worth noting that we do not claim that *R. prolixus* is not able to modulate its responses to heat or to IsobAc after a previous experience. Indeed we do not know if modifying one or some of the training protocols can alter this fact. However, being all the protocols identical except for the conditioned stimulus, we conclude that there is at least a difference in the proneness to respond to the different stimuli after a previous experience. In particular, the two stimuli that were not suitable to become predictors of an unpleasant event *a priori* seem to be the more biologically relevant: heat and alarm pheromone.

Furthermore, the behavioral plasticity observed along this work was not correlated with the innate valence of the stimulus. In this work, *R. prolixus* was innately repelled by an humid substrate, by NaCl and by IsobAc. However, although the intensity of the avoidance behavior generated by the three aversive cues was quite similar, the experience-dependent modulation of such responses was radically different. On the one side, the innate IsobAc avoidance was not suitable to change with our experimental approach. On the opposite side, the innate NaCl avoidance not only disappeared after the training, but gave place to the expression of a new response: insects preferred the side of the arena containing NaCl. In the middle, the innate avoidance of a humid substrate vanished after training, turning into a random spatial distribution. So, we present here evidences that support the idea that the intensity of the experience-dependent modulation of innate negative responses is strongly dependent on the modality of the conditioned stimulus and not on its innate valence.

On the other hand, the two originally neutral stimuli tested in this work elicited a conditioned response after the aversive conditioning. Neutral stimuli are detected by the sensorial system but do not elicit a particular response. In this case, the texture of the substrate or the presence of a dimmed green light did not produce an innate behavior of kissing bugs. However, after training, insects avoided these two originally neutral cues. These results are aligned with the idea that individuals can modulate their innate responses only if the conditioned response is not directly or indirectly harmful for them, as learning to be attracted to a rough substrate or to a green light do not compromise the animal’s survival.

It is worth noting that all experiments were performed in a 2-choice experimental design. In this way, each side of the arena assembled sensory information that guided the insect to a particular spatial preference. For naïve groups, the observed innate preference is the result of the comparison between the valence of the stimuli added at each side. However, during training the negative reinforcement was temporally and spatially coupled with the stimulus added at one side of the arena, for what the final decision of the insect is more complex. Moreover, imagine stimuli “A” and “B,” being neutral. Applying a negative reinforcement associated to “A” can generate two possible processes that could induce learning: (1) A-: an inhibitory one generated by the negative experience of walking over a vibratory substrate with stimulus “A,” or 2) B+: an excitatory one generated by the positive experience of not receiving the vibration when walking over the substrate with the stimulus “B.” In both cases, if learning was to occur, a conditioned avoidance of “A” would be the observed behavioral output. However, the resulting modulation of the innate behavior may arise from the action of one, the other, or the combined action of these two processes. So, a test with an animal spending more time at the “B” side could be due to an acquired repellency to “A,” to an acquired attraction to “B,” or to the combined action of both phenomena. In any case, even if in our work we cannot dissect the exact mechanism (e.g., A- or B+) involved in the experience-dependent modulation of the innate behavior of *R. prolixus*, we unequivocally show that this modulation is dependent on the modality of the conditioned stimulus.

Even if the role of many parameters of a training protocol were shown to be relevant, to our knowledge this is the first work in which the sensory modality of the conditioned stimulus is considered as a modulator of learning processes. Results were quite clear to show that the same conditioning protocol applied together with different stimuli as CS can render very different results, going from not being able to modulate a particular response up to radically change the innate preferences of these bugs. This work enriches the knowledge about cognition processes in arthropods, adding new insights about the behavioral plasticity of an hematophagous insect model. Moreover, taking in consideration that *R. prolixus* is an insect-vector of a human disease and that its DNA has been recently sequenced, it can become a promising model in the learning and memory field. We believe that the experience-dependent modulation of the behavior of these insects should be taken into consideration at the moment of designing control and monitoring field strategies in endemic regions.

## Author Contributions

SM, AC, GP, AM, and NR carried out the experiments. SM performed the statistical analysis. SM and RB designed and coordinated the study and wrote the manuscript.

## Conflict of Interest Statement

The authors declare that the research was conducted in the absence of any commercial or financial relationships that could be construed as a potential conflict of interest. The reviewer MV and handling Editor declared their shared affiliation.

## Supplementary Material

FIGURE S1 Intensity of the negative reinforcement during the operant conditioning. **(A)** The number of vibrations received by *R. prolixus* during trainings (One-way ANOVA, *p* < 0.05) and **(B)** the vibration time received by *R. prolixus* during trainings varied across modalities (One-way ANOVA, *p* < 0.05). Each column represents the mean (±SE) number of vibrations or the vibration time of 30 insects during trainings in a rectangular walking arena. Different letters denote statistical differences between series evinced by Tukey’s comparisons.

FIGURE S2 Correlations between intensity of the negative reinforcement and learning performances. No statistical correlations were found between the number of vibrations or the vibration time and the learning performances of *R. prolixus* (*p* > 0.05 in all cases) of all experimental series. **(A)** Mechanical series, **(B)** visual series, **(C)** thermal series, **(D)** hygric series, **(E)** gustatory series, **(F)** olfactory series.

## Abbreviations

DCM: dichloromethane
IsobAc: isobutyric acid
NaCl: sodium chloride
PI: preference index
